# A handful of inflammation

**DOI:** 10.1016/j.ero.2025.11.013

**Published:** 2025-12-02

**Authors:** Thorben Witte, Fredrik Albach, David Simon, Arnd Kleyer, Robert Biesen, Kay-Geert Hermann

**Affiliations:** 1 Department of Rheumatology and Clinical Immunology, Charité - Universitätsmedizin Berlin, Berlin, Germany; 2 Department of Internal Medicine 3, Friedrich-Alexander-University Erlangen-Nürnberg (FAU) and Universitätsklinikum Erlangen, Erlangen, Germany; 3 Deutsches Zentrum Immuntherapie (DZI), Friedrich-Alexander-University Erlangen-Nürnberg (FAU) and Universitätsklinikum Erlangen, Erlangen, Germany; 4 Deutsches Rheuma-Forschungszentrum Berlin, Berlin, Germany; 5 Fraunhofer Institute for Translational Medicine and Pharmacology, Allergology and Immunology, Berlin; 6 Department of Radiology, Charité–Universitätsmedizin Berlin, Berlin, Germany

## Abstract

Rheumatoid arthritis is an autoimmune disease typically affecting the metacarpophalangeal (MCP) and proximal interphalangeal (PIP) joints as well as larger joints like the wrists, shoulders or knees. We present a case of a 49-year-old woman with symmetrical pain not only in her knees, shoulders, wrists, and MCP and PIP joints but also in her distal interphalangeal (DIP) joints. She has elevated levels of rheumatoid factor and anticitrullinated protein antibodies and a highly active arthritis on magnetic resonance imaging of her right hand in the wrist, MCP, PIP, and 3 out of 4 DIP joints. In summary, we diagnosed a seropositive rheumatoid arthritis affecting all but 1 joint of her hand. This case highlights the very rare (less than 0.2%) affection of 3 out of 4 DIP joints in rheumatoid arthritis.

A 49-year-old female patient presented with symmetrical pain in the knees, shoulders, wrists and metacarpophalangeal (MCP), proximal interphalangeal (PIP) and distal interphalangeal (DIP) joints. There was joint stiffness of up to 3 hours in the morning without tingling in the hands. She had no medical history of psoriasis herself or in her family, but she was a smoker with about 4 pack years in the past.

Upon examination, all described joints were tender with MCP 2 and 3 at both hands being swollen without any sign of dactylitis. She showed an incipient ulnar deviation of fingers 2 to 5 on both sides and complained of a loss of strength in her hands.

The rheumatoid factor (immunoglobulin M 41.1 U/mL; <20 U/mL) was moderately elevated, and the anticitrullinated protein antibodies revealed high positivity (598.6 U/mL; <20 U/mL). No anticell antibodies were detectable in the immunofluorescence on HEp2 cells. The uric acid level was normal. Whipple’s disease was ruled out by polymerase chain reaction from a joint biopsy.

X-ray of the hands detected massive erosive changes in the MCP joints (see Fig 1A). Magnetic resonance imaging of the right hand displayed massive arthritis of the wrist and all MCP and PIP joints, as well as severe arthritis of all DIP joints except for DIP 4 (see Figs 1B-H and Fig 2).

**Figure 1:**
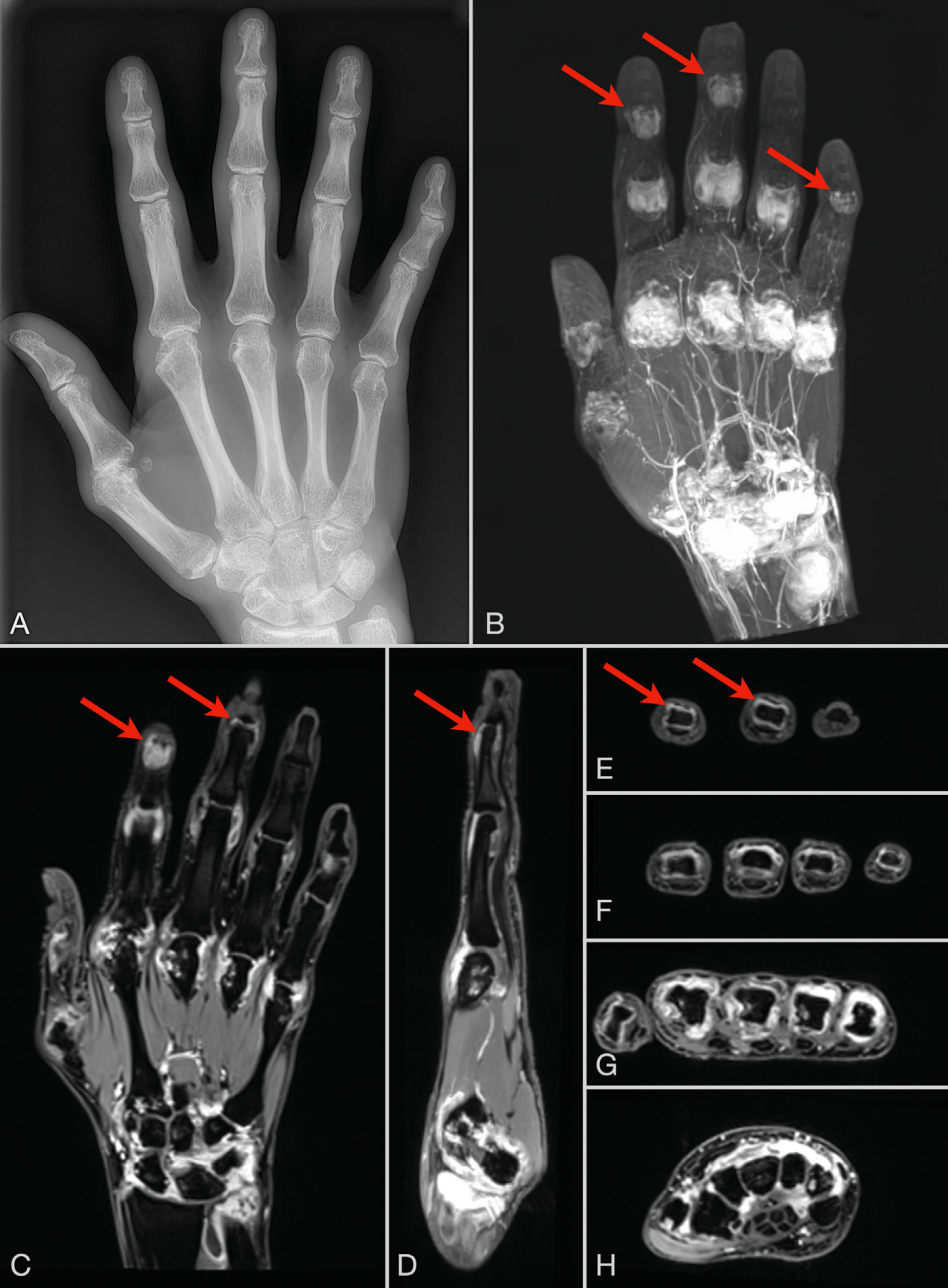
Image of the patient’s right hand with conventional X-ray diagnostics (A) and MRI with contrast agent gadobutrol (B-H). The maximum intensity projection (B) clearly shows all joints affected by synovial inflammation, including involvement of the distal interphalangeal (DIP) joints (red arrows). The individual sections in coronal (C), sagittal (D, 3rd finger), and transverse (E, DIP joints; F, proximal interphalangeal joints; G, metacarpophalangeal joints; H, intercarpal joints) slices confirm the synovial involvement of all joint levels. The red arrows mark the DIP joints.

**Figure 2:**
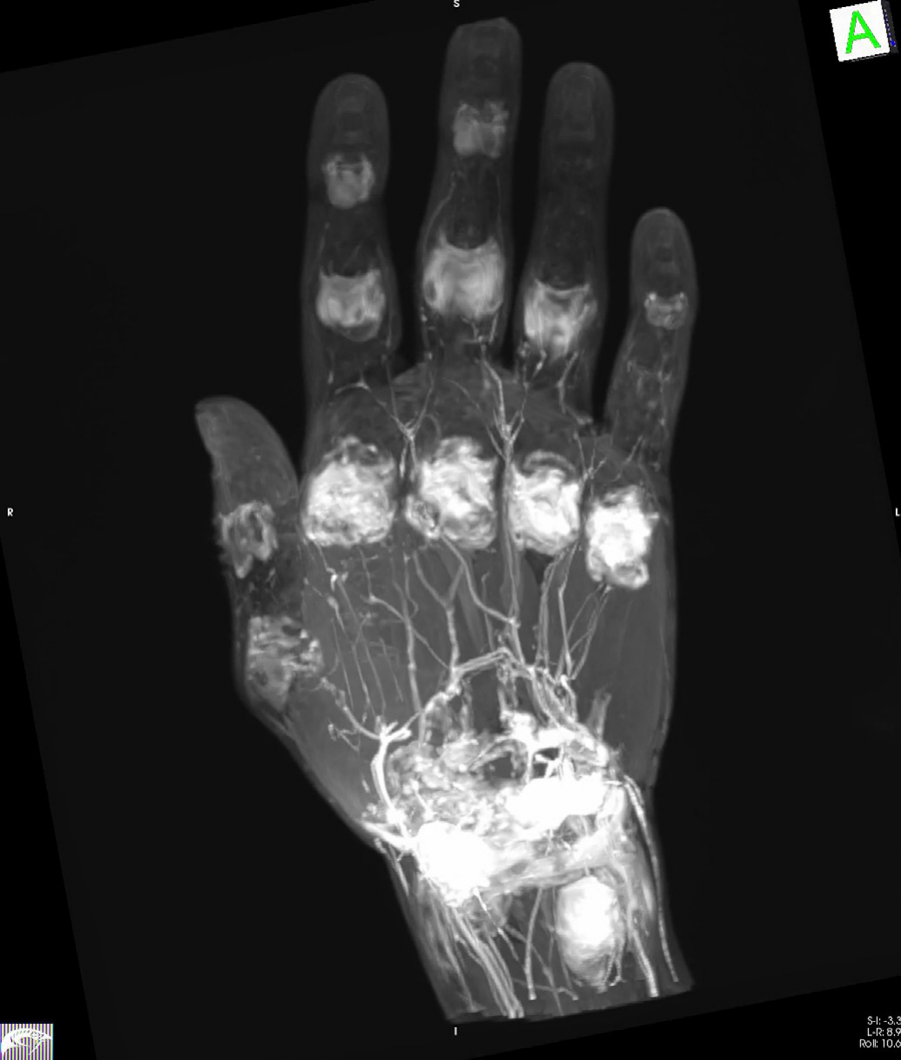
Maximum intensity projection of a magnetic resonance imaging with contrast agent gadobutrol of the patient’s right hand. The video clearly shows all but 1 joint affected by synovial inflammation including distal interphalangeal joint involvement.

In conclusion, we diagnosed a seropositive rheumatoid arthritis (RA) with an elevated disease activity score-28-CRP of 5.21 indicating highly active disease. Inflammation of the DIP joints is a characteristic of psoriatic arthritis, activated Heberden’s osteoarthritis, and is possible in cases of gouty arthritis. RA regularly spares DIPs. Exceptions to this rule, as in this impressive case, are rare and affect approximately 2.1% of all patients with RA. The inflammation of 3 DIP joints in RA as shown here affects less than 0.2% of patients with RA. Like our patient, mainly younger female patients are affected [1].

## Editor disclosure

The peer review process did not involve Editorial Board Members David Simon and Arnd Kleyer, and the editorial decision-making was led by editors who were not involved in the creation of this manuscript.

## Appendix Supplementary materials

Image, video 1 dummy alt text
